# Examining the language demands of informed consent documents in patient recruitment to cancer trials using tools from corpus and computational linguistics

**DOI:** 10.1177/1363459320963431

**Published:** 2020-10-13

**Authors:** Talia Isaacs, Jamie Murdoch, Zsófia Demjén, Fiona Stevenson

**Affiliations:** 1 University College London, London, UK; 2 University of East Anglia, Norwich, UK

**Keywords:** cancer, clinical trials, corpus linguistics, informed consent, research ethics

## Abstract

Obtaining informed consent (IC) is an ethical imperative, signifying participants’ understanding of the conditions and implications of research participation. One setting where the stakes for understanding are high is randomized controlled trials (RCTs), which test the effectiveness and safety of medical interventions. However, the use of legalese and medicalese in ethical forms coupled with the need to explain RCT-related concepts (e.g. randomization) can increase patients’ cognitive load when reading text. There is a need to systematically examine the language demands of IC documents, including whether the processes intended to safeguard patients by providing clear information might do the opposite through complex, inaccessible language. Therefore, the goal of this study is to build an open-access corpus of patient information sheets (PIS) and consent forms (CF) and analyze each genre using an interdisciplinary approach to capture multidimensional measures of language quality beyond traditional readability measures. A search of publicly-available online IC documents for UK-based cancer RCTs (2000-17) yielded corpora of 27 PIS and 23 CF. Textual analysis using the computational tool, Coh-Metrix, revealed different linguistic dimensions relating to the complexity of IC documents, particularly low word concreteness for PIS and low referential and deep cohesion for CF, although both had high narrativity. Key part-of-speech analyses using Wmatrix corpus software revealed a contrast between the overrepresentation of the pronoun ‘you’ plus modal verbs in PIS and ‘I’ in CF, exposing the contradiction inherent in conveying uncertainty to patients using tentative language in PIS while making them affirm certainty in their understanding in CF.

## Introduction

Obtaining informed consent (IC) for research involving participants is an ethical imperative, legal requirement, and widely accepted international standard (World Medical Association, 2013). As a safeguard intended to protect human dignity, welfare, and rights, it necessitates adequate disclosure from investigators about research aims and procedures together with participants’ understanding of the conditions and implications of participation (Silva and Sorrell, 1984). After decades of research on IC, debates centring on fundamental considerations persist, including how much information participants should receive, what constitutes sufficient understanding (Grady, 2015), and how to establish that participation decisions are an act of free will to a sufficient degree (Miller et al., 2009).

One setting where the stakes for understanding are high in view of the potential consequences of research participation is clinical trials, which test the effectiveness and safety of new medical interventions for patients. Much of the IC literature, therefore, resides in the trials methodology research, including randomized controlled trials (RCTs), which are widely regarded as the most robust method for making causal inferences between an intervention and outcome in medicine (Cockayne et al., 2017). However, a perennial challenge is recruiting enough participants, with over half of RCTs failing to meet recruitment targets in some estimates, leading to statistically underpowered studies (Treweek et al., 2018b). Among the recruitment barriers, difficulties related to the IC process have been well-documented (Kearney et al., 2018). The biggest challenge with IC in clinical trials is how to provide meaningful information in a way that potential participants can understand and then use to make a decision about their participation in the study.

Trialists face numerous challenges when communicating ethical information to patients. First, research ethics committees are charged with enforcing processes to protect patients, ensure regulatory compliance, and protect against liability (Beskow et al., 2010). Researchers’ efforts to render IC documents less lengthy and complex are often stymied by ethics committees or project sponsors, presenting obstacles for researchers trying to improve the accessibility of their documents (Grady et al., 2017). Second, patients often have no medical training. Thus, medical concepts need to be explained using plain language, particularly for patients with low literacy, health literacy, and/or language proficiency (Peters et al., 2016). Third, methodological concepts regarding trial design (e.g. ‘trial arm’, ‘placebo’) can be difficult to explain to stakeholders with little background in trials (Tam et al., 2015). Unlike the two aforementioned challenges, this challenge is trial-specific and further contributes to the complexity of the information to be conveyed, particularly for RCTs, due to the need to explain to participants why they are being randomized. But patients are not the only stakeholders who may be unfamiliar with trial design principles. Recruiters themselves vary in their understanding of trial research design features and, hence, in the information they are able to communicate to patients during recruitment consultations (Wade et al., 2017). In sum, trialists are charged with conveying complex specialist information during IC but face barriers to making the information accessible.

Some nested RCTs have examined whether optimizing written IC documents enhances patient understanding and/or recruitment to the host RCT. For instance, Cockayne et al. (2017) compared a control PIS with an optimized version modelled on the National Research Ethics Service template, and another based on user testing and a graphic designer’s input. However, the PIS version that was used had no effect on recruitment. This null result is ambiguous. It could be that when IC is done well (i.e. renders the core information in an understandable and accessible way to prospective participants), this could increase participant recruitment. Conversely, if patients understand more about the conditions and potential repercussions of research participation, this could undermine recruitment efforts—a relationship that needs to be explored in further work but is beyond the scope of the present study. Grady et al. (2017) found that the use of a more concise, simplified CF, compared to a control CF, neither impeded nor improved patients’ understanding of the purpose of randomization or satisfaction with the IC process. However, neither study systematically analyzed language use in the ethical documents beyond unidimensional measures of reading grade level. The current study addresses this gap by analyzing the written discourse of ethical documents, drawing on methods from corpus and computational linguistics to build an evidence base for improving their accessibility.

### Textual analysis in healthcare settings

Textual analysis of medical English using corpus or computational tools, sometimes alongside qualitative methods, has been conducted using medical imaging reports (Friedman, 2000), discharge summaries (Friedman, 1997), medical abstracts (Nye et al., 2018), electronic records (Teufel and Elhadad, 2002), patient information pamphlets (Peters et al., 2016), patient accounts of their experiences (Semino et al., 2018), and patient feedback about health services (Baker et al., 2019). However, textual analysis in trial recruitment research is in its infancy. Few studies have examined the linguistic properties of written IC documents used in trials, and those that do report limited measures such as wordcount and/or readability, which incorporates word and sentence length (e.g. Gillies et al., 2014, although see O’Sullivan et al., 2020, for a wider range of related indices). However, such measures fail to take into account the multicomponential nature of reading that language learning theories and research suggest is not restricted to lexical and syntactic processing, but also encompasses discourse- and semantic-level processing (Koda, 2005). For example, discourse markers and connectives (e.g. ‘but’, ‘however’) provide information about how clauses, sentences, and paragraphs relate to one another, helping the reader grasp how ideas are bound together in extended text. However, the use of such cohesive cues negatively correlates with readability measures because the addition of extra words increases sentence length (McNamara et al., 2014). This aspect of text difficulty has not been considered in trials research and best practice guidelines for crafting ethical documents, which assume that reducing sentence length always improves comprehension (e.g. Health Research Authority, 2019).

Furthermore, word count and readability measures are impervious to meaning. For example, words that are polysemous (i.e. have more than one meaning), such as ‘screening’ or ‘trial’, and especially grammatical words that are both polysemous, and carry relatively little concrete meaning, such as markers of modality (e.g. ‘can’, ‘might’, ‘should’), can increase readers’ processing load compared to content words with only a single sense. The use of polysemous words compounds the challenge of extracting the correct meaning (Laufer, 1997), particularly for patients accessing information in their nondominant language, potentially contributing to communication difficulties (Isaacs et al., 2011). These examples illustrate that text difficulty cannot be adequately characterized using readability alone, which underrepresents the processing involved in reading text (McNamara et al., 2014). A wider array of linguistic measures is needed to capture different facets of text difficulty and language quality.

In light of this research gap and driven by the need to build an evidence base on the language of IC, this mixed methods study draws on techniques from corpus and computational linguistics to investigate the language demands of written IC documents used for RCT recruitment. The goal is to describe the development of the first open-access online corpora of IC documents for RCTs (Isaacs et al., 2019). This is also the first study to systematically compare the linguistic properties of PIS and CF to one another and to two larger reference corpora to examine different facets of text difficulty and language quality by extracting multidimensional measures and analyzing grammatical function.

## Materials and methods

Preferred Reporting Items for Systematic Reviews and Meta-Analyses (PRISMA) guidelines (Moher et al., 2009) were used to select sources to include in our corpora using several criteria. First, we only included RCTs because explaining random allocation could increase language demands (Nishimura et al., 2013). Second, we limited our search to RCTs in progress or completed after 2007, with the timeframe for inclusion January 2007 to July 2017. The UK Medicines for Human Use (Clinical Trials) Regulations came into law in 2004, in line with the EU Clinical Trials Directive (Bollapragada et al., 2007). Therefore, the trials included in our data search fall under this legislative regulation, which aligns with our goal of examining language use in ethical documents for contemporary cancer RCTs. Third, we confined our search to UK-based RCTs targeting any type of cancer for adult patients (⩾ 18 years) who been diagnosed with cancer or were undertaking cancer screening or testing. Nested studies not directly testing cancer interventions and emergency interventions were excluded. We focused on cancer because it affects a wide cross-section of society and receives the largest proportion of UK research funding for any disease type (UK Clinical Research Collaboration, 2020). The expediency of using ‘cancer’ as a search term was another consideration, as we believed that the different types of cancer and interventions would contribute to the breadth of IC documents that we could access. This also enabled us to compile corpora that are sufficiently homogeneous to examine language complexity without interference from factors such as topic or language variety. Finally, for inclusion in our corpora, an English language PIS and/or CF needed to be publicly available online at the time of the data search.

After consulting a medical librarian, we used the search terms ‘Randomised Controlled Trials’ AND ‘Cancer’ as a Health Research Classification System Category, a UK Clinical Research Collaboration Category, or keyword. Identified RCTs were screened against the inclusion criteria using the following e-repositories or databases: (1) National Institute for Health Research (NIHR) Journals Library, (2) Europe PubMed Central for RCTs funded by Cancer Research UK, Prostate Cancer UK, Academy of Medical Sciences, Breast Cancer Now, Breast Cancer Campaign, or Dunhill Medical Trust, (3) Research for Patient Benefit (RfPB) funded studies, (4) Medical Research Council (MRC) funded studies, (5) Medline, (6) Embase and (7) Cochrane Central Register of Controlled Trials.

The search yielded a list of ongoing or completed studies or publications. We collected electronic records of initially eligible studies in a spreadsheet and screened abstracts using the inclusion criteria. In full-text screening, we manually reviewed the contents page, methods, appendixes and supplementary files for IC documents, resolving any eligibility uncertainties through discussion. We searched the ISRCTN registry, ClinicalTrials.gov, and EU Clinical Trials Register for web links where ethical forms might be stored and performed free text searches for ‘consent’, ‘information sheet’ and ‘information leaflet’. Data saturation was then checked against the first 100 Google Scholar entries and RCTs categorized under ‘cancer’ on the Online Resource for Recruitment research in Clinical triAls (ORRCA) database (Kearney et al., 2018). No new sources were identified, suggesting that data sources were exhausted. After removing duplicates, we uploaded citation information of included studies to Endnote X8 and recorded metadata about each associated RCT in a spreadsheet, including study start date, duration, research design information (individual/cluster RCT; number of study arms), patient blinding to the treatment group to which they were assigned, number of interventions and whether they were clinical (e.g. chemotherapy), behavioural (e.g. exercise regimen), or educational (e.g. dietary advice), cancer type(s), and reason for the study (e.g. screening). We also captured information about the ethical documents retrieved, including data source, translation availability, and presence of nontextual information (e.g. flowcharts, tables, other media/formats).

To prepare the PIS and CF corpora for analysis and subsequent digital archiving, each file was converted to .txt format, corrected for spacing/hyphenation, and anonymized. The word ‘TABLE’, ‘DIAGRAM’ or ‘IMAGE’ was inserted in the place of tabular or graphical information. The files were then aggregated to create separate corpora for PIS and CF and deposited in the UK Data Service’s open-source repository, ReShare, along with metadata (Isaacs et al., 2019; see online Supplementary Materials for further information about the search strategy and data preparation).

### Data analysis

The PIS and CF were analyzed separately because they comprise different functions—information provision to help patients make a participation decision in the former, and confirmation that they have understood the conditions and consented to participate in the latter (Health Research Authority, 2019). Coh-Metrix 3.0 (2017), a computational tool extensively used in language research, was used to generate automated measures theorized to align with the processes, structures, and representations involved in processing text. We first report word count and Flesh-Kincaid grade level, a common readability measure, in line with previous trials methodology research. To provide a multidimensional view of textual quality, we report five of what the Coh-Metrix developers coined ‘easability’ dimensions, hereafter referred to as text ease dimensions. These dimensions are the five principal components that an earlier Coh-Metrix validation study had revealed most robustly capture textual differences across text genre and pre-graded level (Graesser et al., 2011). Text ease is, therefore, operationalized here as the overall profile of the five following dimensions:

1. Narrativity: The extent to which the text communicates a story, event, or procedure in conversational style. This dimension is underpinned by word familiarity and given information that links to readers’ prior knowledge. Notably, 17 Coh-Metrix measures in Graesser et al.’s validation study loaded onto the narrativity dimension (component score) as primary measures, contributing to its complex, multi-faceted nature. Narrativity was found to be the most robust dimension in accounting for differences between text genre and grade level. Informational texts about unfamiliar topics that do not resemble oral language would score low on this dimension.
2. Syntactic simplicity: The extent to which sentences are syntactically simple and easy to process. Long sentences with embedded clauses that place demands on readers’ working memory would score low on this dimension.
3. Word concreteness: The extent to which content words in the text are concrete (i.e. have physical form) and imageable (i.e. invoke mental images). Texts laden with abstract concepts would score low on this dimension.
4. Referential cohesion: The extent to which content words and ideas overlap across sentences and the whole text, enabling readers to draw interconnections between them. Texts with little overlap that do not show how different threads relate to one another would score low on this dimension.
5. Deep cohesion: The extent to which the text contains the following categories of connectives to hold the text together: causal (e.g. ‘due to;’ ‘therefore’), temporal (e.g. ‘during;’ ‘finally’), logical (e.g. ‘if’, ‘therefore’), and additive (e.g. ‘in addition;’ ‘furthermore’). Texts with few such connectives would score low on this dimension.

It is beyond the scope of this article to describe how the measures comprising these dimensions were computed. Our purpose is simply to describe the dimensions so that the text ease profiles can be interpreted. We report Coh-Metrix indices in relation to the Touchstone Applied Science Associates (TASA) corpus, the most comprehensive corpus of graded US educational texts, which approximates average American college students’ textual exposure during their lifetime (Jones, 2006). Clearly, the purpose of PIS and CF is different than science texts; however, there are parallels in needing to explain sometimes technical information to a lay audience. There are no medical information texts written for patients, to our knowledge, that are benchmarked to school grade level and, hence, aligned to an expected reading or text difficulty level, making the TASA corpus the best available means of comparison. Previous health research has found that the readability of ethical documents for patients far exceeds the average reading level of the average American, which is considered to be at or below eighth grade level (Eltorai et al., 2015). The American Medical Association (AMA) recommends that written health materials not exceed a sixth grade reading level (Weiss, 2003), whereas the National Institutes of Health (NIH) recommends maintaining a seventh to eight grade level (2017). This guidance made it meaningful to compare the PIS and CF corpora to the TASA science texts at levels approximating AMA and NIH recommendations (grades 6–8) and far exceeding it (grades 11+). We report text ease dimensions as mean percentiles, with higher scores implying less cognitive effort in processing the text.

To complement these analyses, we used the web-based corpus tool, Wmatrix4 (Rayson, 2008), which facilitates running automatic searches and drawing comparisons between electronic corpora, to determine the characteristic lexical and grammatical features of the IC documents, extracting examples from our corpora to illustrate language use. We compared the PIS and CF to a larger general written corpus, Baker’s (2009) million-word British English 2006 (BE06), to investigate differences in lexical frequency. BE06 represents the kind of language that an ‘average’ British-born speaker might use or encounter in general, making it useful for examining lexical and grammatical features that are overrepresented in PIS and CF compared to general written English. We interrogated the data for keywords, key parts-of-speech (POS), and concordances (see McEnery and Hardie, 2012), each of which we describe in the Results section

## Results

**Figure 1.**
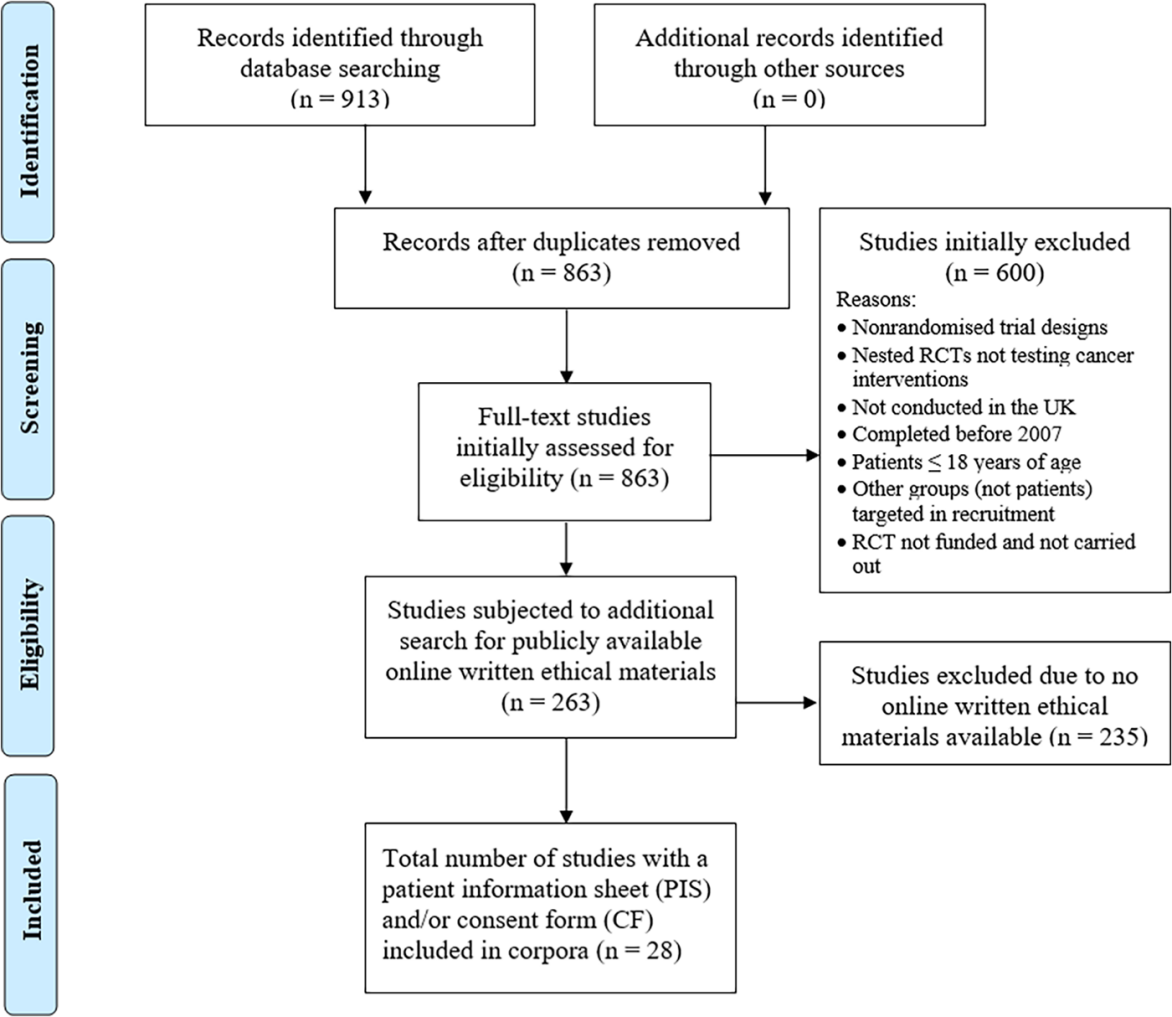
PRISMA diagram summarizing the selection of eligible IC documents for corpora building.

The initial search yielded 863 records after removing duplicates, which were then screened for the inclusion criteria, resulting in 263 RCTs before the criterion of the availability of the PIS or CF online was applied. This resulted in a 62,030-word corpus of 27 PIS and an 8118-word corpus of 23 CF drawn from 28 RCTs (see Figure 1). Twenty-six RCTs randomized patients at the individual level, whereas two were cluster RCTs (Davies et al., 2015; Kitchener et al., 2016). Patients were aware of which treatment they would receive in 26 RCTs, with two blinding patients to the study arm (Langley et al., 2014; Stein et al., 2016). Clinical or procedural interventions were by far the most common (e.g. colonoscopy; Barr et al., 2009) and were a feature of 24 of the 28 RCTs. Eight of the 28 RCTs included at least one behavioural or educational intervention (e.g. healthy eating and physical activity program to promote behaviour change; Koutoukidis et al., 2016). This was coupled with a clinical/procedural intervention in two studies (Davies et al., 2013; Halligan et al., 2015). Only Warde et al. (2012), which recruited patients from multiple countries, gave participants the option of IC documents in a language other than English. Table 1 summarizes further RCT characteristics.

**Table 1.** Characteristics of 28 RCTs whose ethical materials were included in the corpora.

Study^a^	Start date	Type(s) of cancer/tumour	Intervention	Cancer screening	Materials obtained	Data source
Barr et al. (2009)	2009	Oesophageal	Clinical	Yes	PIS; CF	NIHR protocol
Bayman et al. (2016)	2012	Lung	Clinical		PIS; CF	Supplementary material to protocol
Clive et al. (2015)	2011	Lung	Clinical		PIS; CF	Supplementary material to protocol
Davies et al. (2015)	2015	Any type	Clinical		PIS	Supplementary material to protocol
Davies et al. (2013)	1995	Breast	Clinical; behavioural		PIS; CF	Supplementary material to results
Dunn et al. (2013)	2013	Breast	Clinical		PIS; CF	Trial website
Faivre-Finn et al. (2016)	2005	Lung	Clinical		PIS; CF	Supplementary material to protocol
Field et al. (2016)	2014	Lung	Clinical		PIS	Trial website
Foxtrot Collaborative Group (2012)	2007	Bowel	Clinical		PIS; CF	Trial website
Glazener et al. (2011)	2004	Prostate	Behavioural; educational		PIS; CF	NIHR final report
Halligan et al. (2015)	2004	Bowel	Clinical; behavioural		PIS; CF	NIHR final report
Hamdy et al. (2015)	2015	Prostate	Clinical		PIS	Trial website
Hill et al. (2016)	2017	Bowel	Clinical		PIS	ISRCTN Registry
Hubbard et al. (2016)	2013	Bowel	Behavioural; educational		PIS; CF	NIHR final report
James et al. (2016)	2007	Prostate	Clinical		PIS; CF	NIHR final report
Jenkinson et al. (2014)	2014	Meningioma (brain tumour)	Clinical		PIS; CF	Trial website
Kitchener et al. (2014)	2001	Cervical	Clinical	Yes	PIS; CF	NIHR final report
Kitchener et al. (2016)	2011	Cervical	Behavioural; educational	Yes	PIS; CF	NIHR final report
Koutoukidis et al. (2016)	2015	Uterine	Behavioural; educational		CF	Supplementary material to protocol
Langley et al. (2014)	2015	Breast; colorectal; gastrooesophageal	Clinical		PIS; CF	NIHR protocol
Maughan et al. (2013)	2013	Prostate	Clinical		PIS; CF	NIHR protocol
Mulvenna et al. (2012)	2007	Bowel	Clinical		PIS; CF	Trial website with protocol
Primrose et al. (2004)	2004	Lung	Clinical; educational		PIS; CF	NIHR final report
Stein et al. (2016)	2012	Bowel	Clinical		PIS; CF	NIHR final report
Turnbull et al. (2010)	2001	Breast	Clinical		PIS; CF	NIHR final report
Warde et al. (2012)	1995	Breast	Clinical		PIS; CF	Supplementary material to protocol
Williams et al. (2011)	2003	Breast	Clinical		PIS; CF	NIHR final report
Woods et al. (2016)	2015	Prostate	Behavioural		PIS	Trial website via ISRCTN

a First author (year).

**Figure 2.**
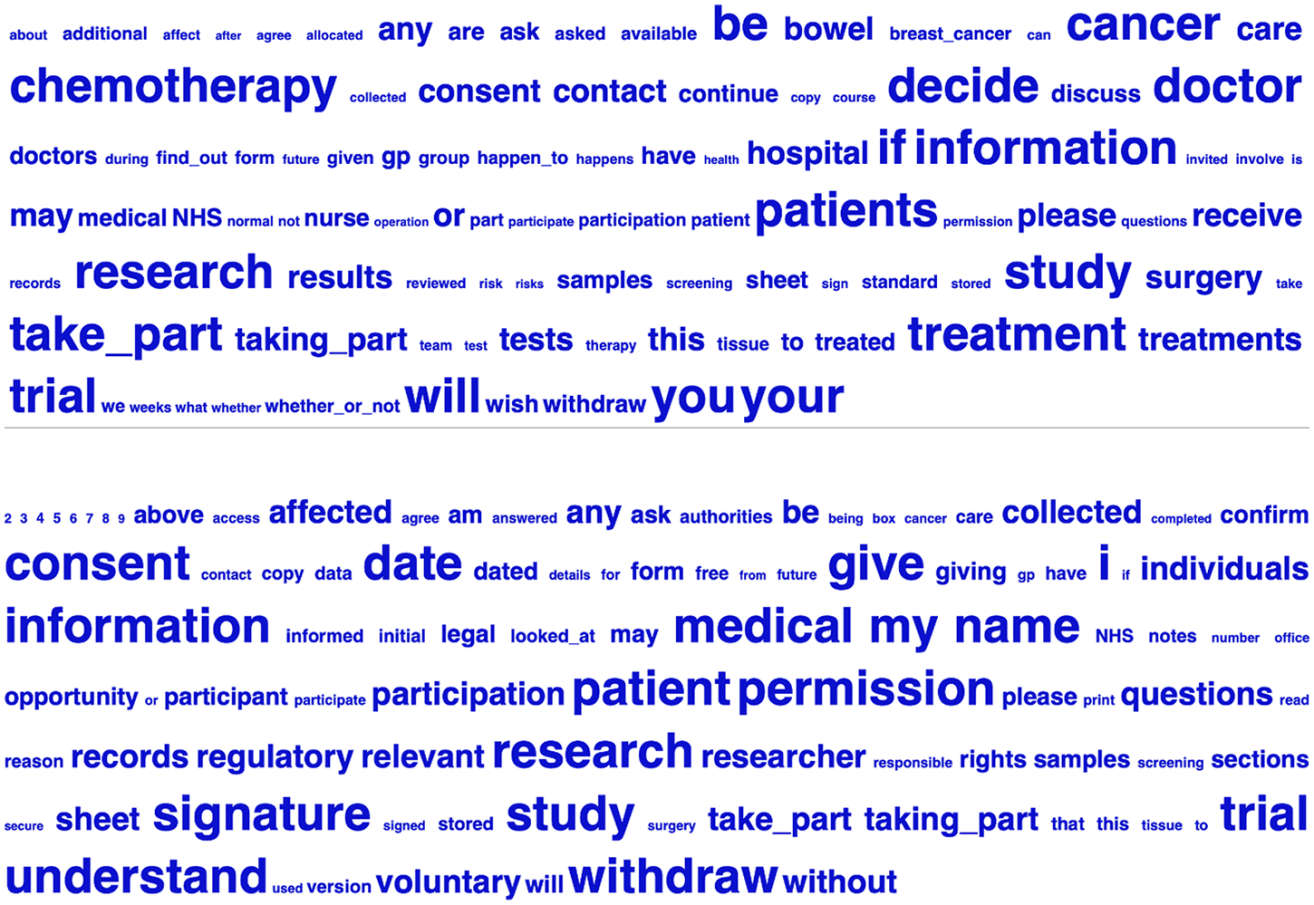
Keyword cloud from PIS corpus (above) and CF corpus (below).

The mean PIS wordcount was 2297.4 (*SD* = 1080.5) compared to 352.6 words for CF (*SD* = 169.9). Eighteen of the 27 PIS were comprised solely of text, six included a trial design flowchart (Foxtrot Collaborative Group, 2012; Hamdy et al., 2015; Langley et al., 2014; Mulvenna et al., 2012; Stein et al., 2016; Woods et al., 2016), three featured tables of study visits or tests and procedures (Faivre-Finn et al., 2016; Hamdy et al., 2015; James et al., 2016), and two included a diagram portraying the condition or intervention (Hamdy et al., 2015; Hill et al., 2016). Figure 2 shows keywords in PIS and CF compared to the BE06 corpus, that is, words that were overrepresented in our corpora compared to the larger reference corpus (BE06) based on log-likelihood (*LL*) to measure statistical significance (*LL* = 10.83, minimum raw frequency of 8, *p* < 0.001; Rayson et al., 2004). We used Log Ratio as an effect size measure and excluded any categories below 1.5, which roughly translates to a feature being more than twice as common in the PIS or CF corpus than in BE06 (Brezina, 2018). The enabled us to focus on the statistically significant categories that represent the largest differences between the corpora while retaining a manageable number of hits.

Notably, ‘cancer’ (‘bowel’, ‘breast’) and ‘chemotherapy’ signal the disease type being targeted in the PIS keyword cloud, and different inflections of ‘treat’ (e.g. ‘treatment’) appear. In contrast, cancer is not prominently featured in the CF keyword cloud, with no reference to (generic/specific) treatment. Therefore, the PIS but not the CF keyword cloud appear to be cancer-specific. Whereas ‘if’, ‘whether’ and ‘decide’ signal uncertainty or condition in the PIS keyword cloud, ‘consent’, ‘permission’, ‘confirm’ and ‘understand’ in the CF keyword cloud imply affirmation.

**Figure 3.**
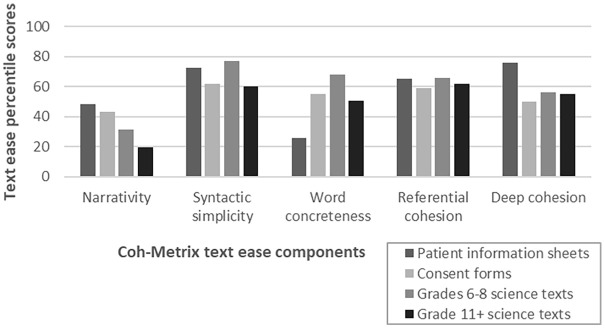
Percentile scores for five text ease components for the PIS corpus, CF corpus, and TASA science texts grades 6 to 8 and 11+.

Mean Flesh-Kincaid grade levels were 9.28 (*SD* = 1.2) for PIS and 9.75 (*SD* = 1.5) for CF compared to TASA science text corpus means of 6.78 for grades 6 to 8 and 10.35 for grades 11+. Figure 3 shows mean percentile scores on the five text ease dimensions for PIS, CF and two TASA science text levels. For narrativity, mean PIS (48.3; *SD* = 10.4) and CF percentile scores (43.2; *SD* =19.3) exceeded TASA science grades 6 to 8 and 11+ (31.5 and 19.7, respectively). This suggests a more story-like quality for the ethical documents than the science texts. In the PIS keyword cloud, for example, keywords contributing to this dimension include storytelling elements such as characters (e.g. ‘you’, ‘patients’, ‘doctor’), setting (e.g. ‘hospital’), and events (e.g. ‘decide’, ‘take part’, ‘surgery’). These words may be more familiar to readers than informationally dense explanations of scientific processes or phenomena (e.g. photosynthesis). Thus, for both ethical genres and particularly for PIS, narrativity positively contributed to overall text ease.

For syntactic simplicity, the mean PIS percentile (72.4; *SD* = 11.4) was slightly lower than TASA science grade 6 to 8 (76.7), whereas the CF percentile (61.7; *SD* = 19.4) was slightly higher than grade 11+ (59.8). That is, the CF is almost equivalent to advanced scientific text on this metric. For example, Hubbard et al.’s (2016) CF, which received the lowest score on the syntactic simplicity dimension, featured the following numbered statement, which the patient needed to initial to indicate consent: ‘I understand that if consent to participate in the study is declined or terminated at any stage, I will enter normal post treatment follow up care’. This 26-word, four-clause long sentence, which expresses the consequence of hypothetical circumstances (conditional tense), is written in passive voice, with no direct indication of the agent who would be declining or terminating participation in the study. The compound words in the noun phrase at the end of the sentence (‘post treatment’; ‘follow up’) are written as separate words without hyphens in the original CF, making it difficult for the reader to parse that these terms are being used as adjectives attributed to the noun ‘case’, particularly because the word ‘treatment’ on its own is more often used as a noun and ‘up’ as a preposition. Primrose et al.’s (2004) CF, which is a more typical exemplar for syntactic simplicity and only slightly exceeds the mean wordcount (62.93), includes the statement: ‘I understand that sections of any of my medical notes may be looked at by responsible individuals from the study group or from regulatory authorities where it is relevant to my taking part in research’. This 35-word sentence is also written in passive voice, contributing to its complexity. The embedded clauses following the word ‘by’ obscure the meaning of who would receive access to patients’ records. It is also unclear which sentence element the pronoun ‘it’ refers to (i.e. ‘sections of any of my medical notes’ accords with the plural pronoun ‘they’), underscoring the difficulty in parsing this sentence.

The mean PIS percentile for word concreteness (25.5; *SD* = 11.7) was markedly lower than the mean for CF (55.1; *SD* = 18.5) and TASA science grades 6 to 8 (67.8) and 11+ (50.7). This suggests that a major source of PIS text difficulty relates to the use of abstract terms (i.e. not detectable using physical senses), including the keywords ‘treatment’, ‘trial’, ‘care’ and ‘participation’. However, abstract concepts are not exclusively used in PIS, as Figure 2 also includes concrete nouns (e.g. ‘doctor’, ‘hospital’). Low word concreteness also contributes to CF text difficulty, although to a lesser extent than for PIS.

PIS received a similar mean percentile for referential cohesion (64.9; *SD* = 13.3) and a higher value for deep cohesion (76.0; *SD* = 12.4) compared to TASA science grades 6 to 8 (67.8 and 55.9 on these dimensions, respectively). This suggests that PIS were written with explicit links between ideas, helping the reader form meaningful connections. For example, this passage from Stein et al.’s (2016) PIS has high referential cohesion due to lexical and content overlap across sentences: ‘You will decide whether or not to continue. If you decide not to continue, your doctor will arrange for your future care. If you do continue, you may be asked to read a new Information Sheet. You might also be asked to sign a new Consent Form’. Conversely, the mean CF referential cohesion (59.0; *SD* = 28.6) and deep cohesion (49.9; *SD* = 24.6) were lower than TASA grades 11+ science texts (61.8 and 54.9, respectively), although the high standard deviations for CF are notable. One explanation relates to the genre of CF as a legal document comprised of declarative statements referring to discrete elements of trial participation expressed as isolated points, with little content overlap across statements. For example, CF clauses, such as ‘I agree to my GP being informed. . .’, ‘I agree to give for this project: tissue samples. . .’, and ‘I understand that I will not benefit financially. . . .’ are written as stand-alone statements with no interlinking. Whereas there are 405 instances of the temporal connectives ‘then’, ‘after’ and ‘during’ in PIS (e.g. ‘If you are harmed due to someone’s negligence, then you may have grounds for a legal action’; ‘During the course of any study it is possible that something may go wrong’), this compares to only 19 temporal connectives in CF, with temporal connectives absent from 10 of the 23 CF. This translates into a lower incidence of temporal connectives for CF (4.3) compared to PIS (13.7), suppressing deep cohesion scores for CF.

**Figure 4.**
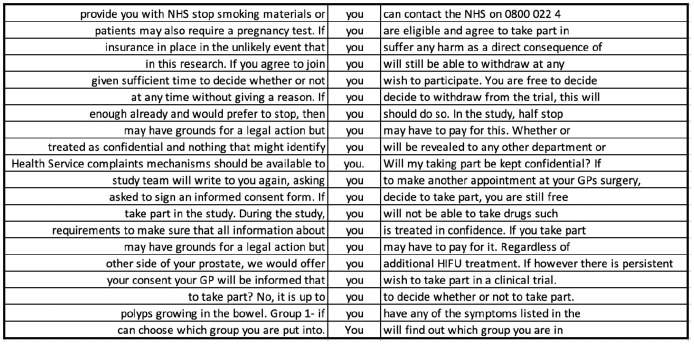
Random sample of 20 concordance lines for the pronoun ‘you’ in PIS corpus.

Next, we used a bottom-up, data-driven approach to examine the grammatical features that characterize the IC materials, extracting examples of language in context (McEnery and Hardie, 2012). To detect significantly more represented POS categories in the ethical corpora compared to BE06 using normalized frequencies (*LL*), we used the same statistical cut-offs as for the keywords above. Log odds (effect size) reveals the odds of the POS category occurring in the PIS or CF corpus compared to the odds of occurrence in BE06 (Brezina, 2018). Table 2 shows that the top key POS category in the PIS corpus compared to BE06 is the polysemous (ambiguous) second-person pronoun ‘you’. Prototypically, ‘you’ refers to one or more addressee(s) in an interaction, but it also performs other functions in English, with its precise referent context-dependent (Quirk et al., 1985). For example, ‘you’ can also be used generically to mean ‘one’, and the referent in this case may or may not include both addressee and speaker. Figure 4 shows a random sample drawn from a concordance – that is, a list of all occurrences of the term ‘you’ from the PIS corpus, with a few words shown before or after. These examples, which demonstrate how the word is used in context, suggest that ‘you’ was used in PIS for different reasons, most frequently to outline what may happen or be offered to the participant, what they, in turn, would do, and any conditions or restrictions that apply.

**Table 2.** Key POS categories for PIS corpus.

POS tag	POS category descriptor	Frequency in PIS		Frequency in BE06		Log-likelihood	Log ratio	Examples
		Raw	Per 1000	Raw	per 1000			
PPY	2nd person personal pronoun (you)	2085	3.56	4796	0.52	3929.9	2.79	*You*
VM	Modal auxiliary (can, will, would, etc.)	2486	4.25	12086	1.3	2214.23	1.71	*will, can, may*
VBI	Be, infinitive (It will be. . .)	1069	1.83	5297	0.57	927.28	1.68	*be*
DD	Determiner (capable of pronominal function) (e.g. any, some)	533	0.91	2440	0.26	514.41	1.79	*any, some*
CSW31	Whether or_not	49	0.08	23	0	189.58	5.08	*whether or not*
CSW	Whether (conjunction)	119	0.2	423	0.05	153.76	2.16	*whether, if*
NN121	Follow_up as singular noun	39	0.07	35	0	122.36	4.15	*follow up*
VDN	Done	49	0.08	243	0.03	42.45	1.68	*done*

The POS tag in the first column are labels or codes used in Wmatrix’s automated tagging system, which automatically assigns a grammatical category (POS) to keywords in the corpus. The ‘Category descriptor’ in column 2 elaborates what each POS tag refers to.

Approximately 25% of the instances of ‘you’ in PIS (502/2083) were immediately preceded by ‘if’ (a keyword). Nearly a third were immediately followed by a modal verb (675/2083), itself the second key POS category, with the formulaic phrase ‘if you would like’ (39/2083) the only overlap between these two uses of ‘you’. ‘If’ prototypically expresses a conditional and restricts the truth value or certainty of a statement. Similarly, modal verbs tend to express different degrees of certainty or obligation. Certainty that is restricted with ‘if’ is reasonably clear to interpret (Y happening is predicated on X happening first), although, as a complex sentence with at least one dependent clause, it increases readers’ cognitive load (McNamara et al., 2014). The same is true of ‘whether’ when used as a conjunction, which is sixth in the key POS list. However, inferring the meaning of modal verbs such as ‘can’, ‘may’, and ‘might’ is less straightforward. These are polysemous (e.g. ‘can’ expresses possibility/probability, ability, and permission) and denote variable degrees of certainty as well as ability and possibility, which are not fixed and, therefore, may be difficult to interpret (Quirk et al., 1985). These different shades of meaning are likely to substantially increase PIS language demands.

The significantly higher use of modal verbs in PIS compared to the BE06 reference corpus implies that readers’ grasp of the range of meanings that they are able to express is important. The most frequently used modal verb in PIS is ‘will’, accounting for 57% of all modal verbs (1414/2486). The next most frequent modal verbs were ‘may’ (380/2486), ‘can’/‘cannot’ (273/2486), and ‘would’ (230/2486), with some uses of ‘should’, ‘might’, ‘could’, ‘must’ and a handful of instances of ‘shall’. ‘Will’ is most frequently used to describe how the trial will be conducted, including procedures, documentation, confidentiality, results, dissemination and treatment or test administration. In these instances, ‘will’ expresses the highest possible level of certainty about some future event (e.g. ‘you will be asked to complete a number of questionnaires’, ‘your remaining samples will be destroyed’). Less certain modal verbs, such as ‘may’, generally describe procedures or outcomes that may not apply to all participants or depend on certain circumstances (e.g. ‘side effects are listed below, but you may or may not have these’). However, ‘may’, ‘would’, and ‘might’ are also used to describe more concretely established procedures (e.g. ‘we may collect some information from your hospital notes or NHS [National Health Service] records’). Less certain modal verbs are also typically used to explain the risks of participation, and the range of modal verbs used in this way could be confusing. For example, modal verbs in ‘your blood pressure may also fall’, ‘cisplatin can affect your kidneys’, ‘tamoxifen might also increase the risk of’, and ‘taking part in this study may result in added costs to me’ express different degrees of certainty but not on a fixed, clearly interpretable scale. A potential participant may, therefore, have difficulty differentiating between risks and their likelihood of occurrence. Some PIS counteract the vagueness of modal verbs (Cutting, 2007) by providing statistics alongside or instead of uncertain modal verbs (e.g. ‘between 1 in 10 and 1 in 100 people will experience these side effects’), although interpreting such figures would assume a degree of numeracy on the part of the patient (Academy of Medical Sciences, 2017). Less certain modal verbs are also often used to describe what the objectives or benefits of the trial will be (e.g. ‘it is believed that covered stents may be more effective’, ‘it may benefit others taking part’, and ‘to see if people recovering from bowel cancer can also benefit’). Such hedging could obscure participation benefits for patients. However, other PIS do opt for more definite benefit statements using the modal verb ‘will’, such as ‘the results of this research will be used by the NHS to decide’ and ‘this way we will be able to find out which works best’. ‘In addition, modal verbs are not all equal in terms of frequency in English generally, including ‘may’, which tends to be less frequent than ‘will’ (also proportionally represented in the corpora). Therefore, some modal verbs are both more ambiguous, and less likely to have been encountered by people for whom English is not a dominant language (Nation, 2013).

**Figure 5.**
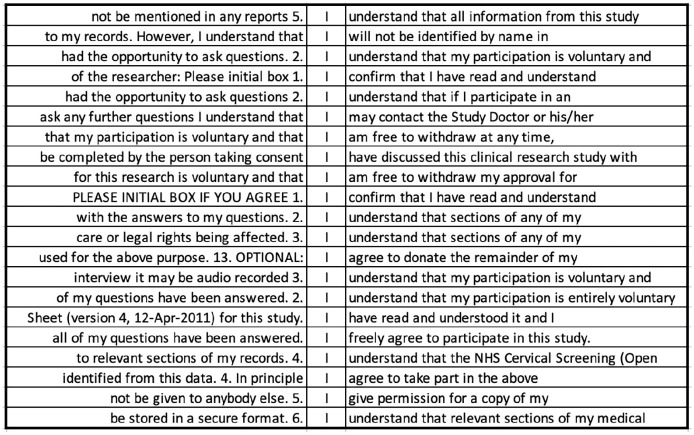
Random sample of 20 concordance lines for the pronoun ‘I’ in CF corpus.

The top key POS category in CF is also a pronoun—the first person ‘I’ (see Table 3). Thus, when PIS and CF are considered together, two different pronouns are used to denote the same referent, namely the participant. In fact, even within the CF corpus, both ‘I’ and ‘you’ occur with reference to the participant (although ‘you’ does not occur at a statistically significant level of frequency), which could breed confusion. In CF, ‘I’ is frequently used in word combinations such as, ‘I am free to withdraw’, ‘I confirm that’, ‘I have read and understood’, ‘I give (my) permission’, ‘I agree to’, and ‘I understand that’ (see Figure 5). Such uses account for almost 70% of the uses of ‘I’ (239/353). What is interesting about these phrases is their unmitigated nature. There are a few instances of modal verbs following ‘I’, but these are mainly ‘will’, which expresses high certainty, and a handful of instances of ‘may’ and ‘can’, in this case denoting permission or ability rather than limiting certainty. In contrast to PIS, where varying degrees of certainty and conditionals characterize the immediate co-text of ‘you’ (i.e. the participant being referred to), in CF, absolute certainty characterizes the context of the participant-referring expression ‘I’. While the information that the patient is given is tentative and hedged in PIS, he/she can only choose to confirm certainty of understanding the conditions of the trial in CF, potentially following oral requests to clarify information, with the only alternative being to not complete the form and, thereby, withhold consent. Reconciling these two contradictory positions in which a potential signatory is being cast is likely to increase cognitive load and may be jarring for some, potentially deterring participation.

**Table 3.** Key POS categories for CF corpus.

POS tag	POS category descriptor	Frequency in PIS		Frequency in BE06		Log-likelihood	Log ratio	Examples
		Raw	per 1000	Raw	per 1000			
PPIS1	1st person singular pronoun (I)	353	4.55	7717	0.83	613.3	2.45	*I*
CST	That (conjunction)	180	2.32	7292	0.78	150.17	1.56	*that*
VBI	Be, infinitive (It will be. . .)	142	1.83	5297	0.57	134.03	1.68	*be*
DD	Determiner (capable of pronominal function) (e.g. any, some)	84	1.08	2440	0.26	109.15	2.04	*any, some*
VH0	Have, base form (finite)	77	0.99	2809	0.3	75.01	1.72	*have*
VHN	Had (past participle)	25	0.32	304	0.03	67.86	3.3	*had*
VBM	Am	34	0.44	821	0.09	53.74	2.31	*am*
VBG	Being	31	0.4	897	0.1	40.46	2.05	*being*

The POS tag in the first column are labels or codes used in Wmatrix’s automated tagging system, which automatically assigns a grammatical category (POS) to keywords in the corpus. The ‘Category descriptor’ in column 2 elaborates what each POS tag refer to.

## Conclusion

This mixed methods study describes the development of the first open-access online PIS and CF corpora for RCTs (Isaacs et al., 2019). It demonstrates the potential of analyzing textual data using underutilized corpus and computational tools in trials methodology research. This interdisciplinary approach could lead to new ways of examining language use and textual difficulty in ethical documents across social science and medical domains. The results confirm previous findings showing that CF fail to meet recommended readability levels for public health documents (Eltorai et al., 2015) and extend them to PIS. Moving beyond unidimensional readability measures, Coh-Metrix text ease profiles reveal that for PIS, the major source of discourse-level text difficulty lies in low word concreteness. This poses a challenge because core IC concepts (e.g. ‘take part’, ‘withdraw’, ‘treatment’, ‘participation’, ‘permission’) and aspects of trial design (e.g. randomization) are abstract and may need to be carefully constructed in text to cater to all patients. The linguistic dimensions that detract from text ease are more distributed across dimensions for CF than for PIS. CF syntactic simplicity and word concreteness values are slightly higher than in the advanced scientific reference text, whereas referential and deep cohesion scores are slightly lower. The Health Research Authority’s (2019) guidelines not to ‘use the passive voice’ nor to ‘introduce more than one idea/point in a sentence’ for PIS should also extend to CF, as more complex sentences can heighten readers’ processing load (Graesser et al., 2011).

Although appearing to render CF more difficult, a counter explanation for the low referential and deep cohesion scores is that these dimensions are not relevant to the CF genre due to the lack of overlap between ideas and absence of causal relationships. This is reflected in the overall structure of CF, which consists of discrete (often numbered) statements expressing different conditions of participation with little content overlap across statements. Future research could consider whether other measures/dimensions of textual difficulty are more germane while also examining interrelationships among the examined indices to empirically establish potential trade-offs or back researchers’ claims that optimized versions of IC documents are, in fact, improved on all metrics.

At first glance, the high PIS and CF narrativity scores may appear surprising. In light of the composite measures that comprise the narrativity dimension, this finding can be partially explained by the overarching focus on individuals as characters in a story, including the extensive use of the pronouns ‘I’ and ‘you.’ In addition, in the two keyword clouds for both ethical genres, ‘patient(s)’, ‘doctor’, ‘researcher’, ‘participant’, ‘nurse’, name the actors in the story, whereas ‘hospital’ and ‘office’, reveal the setting, underscoring the human element and setting the scene for action. This would positively contribute to narrativity compared to discussing scientific concepts or processes, particularly that refer to inanimate objects or phenomena removed from everyday lay conversation. Although these storyesque elements are clearly present, the narrativity algorithm is impervious to which concepts and, by extension, words are related to the most important elements of IC and which are not. For example, patients’ understanding of key concepts such as ‘treatment(s)’, ‘consent’, ‘withdraw’, ‘samples’, ‘the study’ and ‘voluntary’ are arguably fundamental to IC, but their importance relative to other terms is not reflected in the percentile score. Further, some of these words are polysemous and it is not clear how the algorithm deals with their semantic meaning. For example, it may be that ‘study’ is interpreted by the algorithm to mean a room at home where work can be done (as opposed to the intended meaning of a research investigation), ‘treatment’ is regarded as how a person is treated (as opposed to the intended meaning of medical treatment or experimental treatment), and ‘trial’ is assumed to be a legal trial (rather than one conducted in healthcare settings). The secondary meaning of these terms as they are used in IC would likely mean that the narrativity percentile scores are artificially inflated relative to what they should be if the algorithm took into account the correct, less familiar sense of the term.

Taken together, our analyses suggest that text difficulty involves more than word count or readability. For example, such measures are impervious to differences in word concreteness that could markedly affect the linguistic complexity of texts and, in turn, how easy they are for readers to process and understand (Košak-Babuder et al., 2019). Burman et al. (2003) reveal that ethics review committees sometimes mandate that ethical forms be rewritten if they do not achieve a certain readability level but that this is counterproductive, leading to lengthier documents with more textual errors. Overemphasizing readability at the expense of other aspects of text difficulty would seem to be underrepresenting this multifaceted construct, including in studies testing the efficacy of using reportedly optimized IC documents (Beskow et al., 2010) or best practice guidelines for writing ethical forms (e.g. National Institutes of Health, 2017).

The key POS analysis reveals a fundamental contradiction in how (un)certainty is expressed in PIS (e.g. you + modal verb) versus CF (e.g. I + verb of affirmation + that). Although often operationalized as separate genres, the PIS and CF work together. They position participants in the contradictory position of being, in the PIS, uncertain about the intervention, conduct of the trial, or consequences of participation, while in the CF, requiring them to demonstrate certainty in their understanding, which underpins their participation decision. This raises questions about how uncertainty and risk need to be communicated to potential trial participants and what this means for patients when they need to sign against legally binding declarative statements that confirm their understanding of what participation entails in CF (Nishimura et al., 2013). This issue is particularly pertinent when the stakes for participation are high, as is often the case in cancer trials (e.g. invasive treatments, difficult side-effects, intervention may or may not prolong lives; Davies et al., 2015).

A 2017 Academy of Medical Sciences report argues for redressing the balance in communicating risks and benefits in patient information leaflets accompanying medication, claiming that risks tend to be overemphasized and potential benefits insufficiently highlighted. Notably, trials are a different context (e.g. participant recruitment pressures), although there are some parallels with drug leaflets (e.g. lay audience). The use of hedging—that is, vague, tentative language using less certain modal verbs—could dissuade patients from participating. Being more definite about benefits of the study using the modal verb ‘will’ could mitigate this. However, doing so may be misleading when there is uncertainty about how individual patients will react to an intervention (e.g. group aggregate effects cannot predict individual outcomes; Academy of Medical Sciences), unless the benefit being emphasized is about altruistic good in improving knowledge for the benefit of science, society, or others with the disease rather than the effect of the treatment (whichever is assigned) on the individual. That is, the onus is on the researchers to present the benefits of trial participation as truthfully as possible, which could mean not framing benefits in definite terms, although this could lead to prospective participants’ lower comprehension of the degree of risk involved. There are also instances of researchers attempting to minimise risk in PIS using ‘will’ or the simple present to convey certainty (e.g. ‘this does entail some risk, but in this case the benefits outweigh any such risk’; ‘there will be no additional radiation risk from you taking part in the trial and you are not likely to suffer’). In most cases, risk level is written in vague or relativistic terms, making decisions about participation based on risk assessment difficult. It may be that quantifying risk using simple statistics in context is not always possible, and patients’ ability to interpret them may also be an issue. We wish to advance the idea of using corpus extracts (e.g. context-laden concordance lines) to gauge patient preference for the way that concepts such as risk/benefit or randomization are communicated in future research, in conjunction with different measures of their understanding. This would build the evidence base for optimizing how IC documents are crafted, buttressing language-based best practice recommendations in ways that accord with patients’ perspectives (e.g. Health Research Authority, 2019).

### Limitations

This study has several limitations. First, our corpora only include a small number of publicly available materials located through online database searches so that we could make the resulting corpora available open access. Exemplars or extracts from such open access IC repositories could be randomized and embedded within a larger host trial to see which wording is most effective (Treweek et al., 2018a). However, increasing corpora size is essential in future research so that more robust characterizations of the language of IC can be obtained. This could enable investigations of trends over time, comparisons across countries/regions, and differences across medical conditions, for example. Second, we did not validate Coh-Metrix easbility dimensions, which were robust in determining differences in previous research (Graesser et al., 2011), for use with the ethical genres in this study. Future research could probe whether other measures are more appropriate for characterizing textual differences in ethical documents. Third, to inform the direction of our study, we ran patient and public involvement sessions with six volunteers (cancer patients, carers, policy reviewers) who guided us in an advisory capacity. However, their voices and those of other stakeholders (e.g. recruiters, ethical reviewers) are not directly reflected in this paper. Fourth, although assumptions about patient understanding underpin this study, this construct was not examined. Future research could investigate objective and subjective measures of patient understanding in conjunction with textual analyses (see Gillies et al., 2018). Fifth, the comparator corpora used here are not specific to health or legal domains, nor to the language of research ethics. Future research could use other reference samples, including medical or legal corpora. Next, although six PIS contained nontextual information, we excluded these data and solely examined textual information in our analyses. Future research could employ multimodal analysis to examine all sources of nontextual information (e.g. flowcharts, videos), potentially in conjunction with textual analyses of IC conversations, to capture all forms of communication provision available to prospective participants (Wade et al., 2017). Finally, proposing concrete best practice guidelines for crafting IC documents would be premature based on the limited evidence generated in this study, including because patient representatives were not directly consulted. However, there are steps that would enable us to do so in an evidence-based way in future research. As per the above, we would suggest drawing on examples from larger-scale corpora to elicit different indicators of prospective participants’ understanding of alternative framings of similar concepts. This would need to be paired with research on the best cocktail of linguistic measures (including discourse-level measures) that capture key elements of textual quality for information that is deemed essential for IC, given community or stakeholder consensus of what those core elements are. Taken together, this could begin to provide an evidential basis for genuinely improving information provision in PIS and CF, thereby making participants’ decision-making truly more informed. Clearly, there is fertile ground for applying and extending the methods presented here to better understand the linguistic facets of textual difficulty in research ethics communication.

## Supplemental Material

Electronic_supplementary_material_1 – Supplemental material for Examining the language demands of informed consent documents in patient recruitment to cancer trials using tools from corpus and computational linguistics Click here for additional data file. Supplemental material, Electronic_supplementary_material_1 for Examining the language demands of informed consent documents in patient recruitment to cancer trials using tools from corpus and computational linguistics by Talia Isaacs, Jamie Murdoch, Zsófia Demjén and Fiona Stevenson in Health:

Electronic_supplementary_material_2 – Supplemental material for Examining the language demands of informed consent documents in patient recruitment to cancer trials using tools from corpus and computational linguistics Click here for additional data file. Supplemental material, Electronic_supplementary_material_2 for Examining the language demands of informed consent documents in patient recruitment to cancer trials using tools from corpus and computational linguistics by Talia Isaacs, Jamie Murdoch, Zsófia Demjén and Fiona Stevenson in Health:
