# Supplementary material for: Examining the language demands of informed consent documents in patient recruitment to cancer trials using tools from corpus and computational linguistics
Source: Health (London). 2020 Oct 13;26(4):431–56. doi: 10.1177/1363459320963431 (PMC9163777; doi:10.1177/1363459320963431)
Supplement: Electronic_supplementary_material_1 – Supplemental material for Examining the language demands of informed consent documents in patient recruitment to cancer trials using tools from corpus and computational linguistics [file Electronic_supplementary_material_1.pdf]

**Electronic Supplementary Material 1**

## Search strategy and eligibility information by online platform

NIHR Journals Library
URL: https://www.journalslibrary.nihr.ac.uk

We included all RCTs categorised as ‘Primary Research’ for the following funding streams: Health Technology Assessment, Health Services and Delivery Research, Public Health Research, Programme Grants for Applied Research, and Efficacy and Mechanism Evaluation.

Search strategy: HRCS Category ‘Cancer’

Europe PMC
URL: https://europepmc.org

We included all studies funded by Cancer Research UK, Prostate Cancer UK, Academy of Medical Sciences, Breast Cancer Now, Breast Cancer Campaign, and Dunhill Medical Trust.

Search strategy:
(ABSTRACT:"cancer") AND (GRANT_AGENCY:"Cancer Research UK" OR GRANT_AGENCY:"Academy of Medical Sciences (AMS)" OR GRANT_AGENCY:"Breast Cancer Now" OR GRANT_AGENCY:"Breast Cancer Campaign" OR GRANT_AGENCY:"The Dunhill Medical Trust") AND SRC:med AND HAS_FT:y AND (PUB_TYPE:"Randomized Controlled Trial") AND (LANG:"eng" OR LANG:"en" OR LANG:"us")

Research for Patient Benefit
URL: https://www.nihr.ac.uk/research-and-impact/research/nihr-studies/research-for-patient-benefit.htm

Search strategy: UKCRC Category ‘Cancer’

Medical Research Council
URL: https://www.mrc.ac.uk/research/funded-research

We included all funded RCTs that conformed with our inclusion parameters.

Search strategy: HRCS Category ‘Cancer’

Medline and Embase
URL: http://ovidsp.ovid.com

Search strategy:
‘RCT* AND cancer AND (consent form) AND (information sheet) AND UK’

This search produced 9009 publications from potentially eligible studies. From these results, we manually reviewed the first 5% (450), of which 3.8% (17 out of 450) were studies that had not already

been identified. However, no new PIS or CF were located for these 17 studies for inclusion in our corpora. On that basis, we decided a complete review of the full 9009 was unlikely to substantially result in the identification of new data and searching was ceased.

Cochrane Central Register of Controlled Trials
URL: http://www.cochranelibrary.com/about/central-landing-page.html

Search strategy: RCT* AND cancer AND UK

Google Scholar
URL: https://scholar.google.co.uk

Search strategy: ‘informed consent’ AND ‘comprehension’ AND ‘trials’ AND ‘cancer’

We restricted our search to RCTs published between 2006 and 2017 and manually reviewed the first 100 entries to check for data saturation. This search did not lead to any new CF or PIS for inclusion in our corpora.

ORRCA database
URL: http://www.orrca.org.uk

Search strategy: Searched all abstracts grouped under the health area ‘cancer’

We ordered the resulting studies by year to manually review those published since 2006 as a final check for data saturation. This search did not result in any new CF or PIS for inclusion in our corpora.
