# Supplementary material for: Examining the language demands of informed consent documents in patient recruitment to cancer trials using tools from corpus and computational linguistics
Source: Health (London). 2020 Oct 13;26(4):431–56. doi: 10.1177/1363459320963431 (PMC9163777; doi:10.1177/1363459320963431)
Supplement: Electronic_supplementary_material_2 – Supplemental material for Examining the language demands of informed consent documents in patient recruitment to cancer trials using tools from corpus and computational linguistics [file Electronic_supplementary_material_2.pdf]

Electronic Supplementary Material 2

Procedures for data cleaning and archiving

To prepare the PIS and CF corpora for analysis and digital archiving, the original files were converted to .txt files. Text recognition tools or manually typing text was used in rare instances when the words could not be automatically detected. We then cleaned the data using the following steps:

- Spacing irregularities corrected
- Hyphens splitting single words over two lines or between two words removed (e.g., ‘once-daily’ changed to ‘once daily’)
- Text appearing on the forms but not presented to patients deleted (e.g., publication footnotes)
- Typos and grammatical errors in the original documents and introduced by the conversion process corrected (e.g., ‘principle’ changed to ‘principal’; ‘satified’ to ‘satisfied’; ‘asprin’ to ‘aspirin’; ‘lead’ to ‘led’; ‘you family’ to ‘your family’)
- Nontextual information deleted and ‘TABLE’, ‘DIAGRAM’, or ‘IMAGE’ inserted to demarcate where the original information had appeared
- Researchers’ names, institutional information, and contact details removed; however, RCT name, acronym, and website retained
